# Energy homeostasis genes modify the association between serum concentrations of IGF-1 and IGFBP-3 and breast cancer risk

**DOI:** 10.1038/s41598-022-05496-1

**Published:** 2022-02-03

**Authors:** Rocío Rodríguez-Valentín, Gabriela Torres-Mejía, Louis Martínez-Matsushita, Angélica Angeles-Llerenas, Liliana Gómez-Flores-Ramos, Roger K. Wolff, Kathy B. Baumgartner, Lisa M. Hines, Elad Ziv, Lourdes Flores-Luna, Luisa Ma. Sánchez-Zamorano, Eduardo Ortiz-Panozo, Martha L. Slattery

**Affiliations:** 1 Center for Population Health Research, National Institute of Public Health, 62100 Cuernavaca, Morelos Mexico; 2 Department of Geriatrics, Hospital General Regional 251, 52148 Metepec, Estado de Mexico Mexico; 3 Cátedras CONACYT-Center for Population Health Research, National Institute of Public Health, 62100 Cuernavaca, Morelos Mexico; 4 Department of Medicine, University of Utah, Salt Lake City, UT 84108 USA; 5 Department of Epidemiology and Population Health, School of Public Health and Information Sciences, University of Louisville, Louisville, KY 40202 USA; 6 Department of Biology, University of Colorado Colorado Springs, Colorado Springs, CO 80918 USA; 7 Department of Medicine, Institute of Human Genetics, University of California San Francisco, San Francisco, CA 94158 USA

**Keywords:** Breast cancer, Epidemiology, Genetic interaction

## Abstract

Breast cancer is a multifactorial disease in which the interplay among multiple risk factors remains unclear. Energy homeostasis genes play an important role in carcinogenesis and their interactions with the serum concentrations of IGF-1 and IGFBP-3 on the risk of breast cancer have not yet been investigated. The aim of this study was to assess the modifying effect of the genetic variation in some energy homeostasis genes on the association of serum concentrations of IGF-1 and IGFBP-3 with breast cancer risk. We analyzed 78 SNPs from 10 energy homeostasis genes in premenopausal women from the 4-Corner’s Breast Cancer Study (61 cases and 155 controls) and the Mexico Breast Cancer Study (204 cases and 282 controls). After data harmonization, 71 SNPs in HWE were included for interaction analysis. Two SNPs in two genes (*MBOAT* rs13272159 and *NPY* rs16131) showed an effect modification on the association between IGF-1 serum concentration and breast cancer risk (*P*_interaction_ < 0.05, adjusted *P*_interaction_ < 0.20). In addition, five SNPs in three genes (*ADIPOQ rs182052, rs822391 and rs7649121, CARTPT rs3846659, and LEPR rs12059300*) had an effect modification on the association between IGFBP-3 serum concentration and breast cancer risk (*P*_interaction_ < 0.05, adjusted *P*_interaction_ < 0.20). Our findings showed that variants of energy homeostasis genes modified the association between the IGF-1 or IGFBP-3 serum concentration and breast cancer risk in premenopausal women. These findings contribute to a better understanding of this multifactorial pathology.

## Introduction

Breast cancer is a multifactorial disease in which the interplay among multiple risk factors remains unclear. Some central and peripheral regulators of food intake and energy expenditure (energy homeostasis) foster cancer development^1^. Dysregulated energetic metabolism has been strongly associated with breast cancer (BC) risk^1, 2^. In addition to their role in food intake or fuel metabolism, energy homeostasis factors regulate tumorigenic processes. Central regulators such as the hypothalamic orexigenic neuropeptide Y (NPY) and anorexigenic cocaine and amphetamine regulated transcript (CART) peptide have been involved in different kinds of cancers^3, 4^. NPY promotes cellular proliferation, invasion, metastasis and angiogenesis through its different receptors^3^, and the CART peptide is expressed in primary and metastatic BC cells^4^. Among peripheral regulators, leptin (LEP) signaling stimulates proliferation, survival, migration and cell invasion in BC^5–7^, and adiponectin (ADIPOQ) inhibits proliferation and metastasis of BC cells^5, 6^. The ghrelin (GHRL) peptide has been associated with increased proliferation of BC cellular lines^8^. The enzyme ghrelin-O-acyltransferase (GOAT or MBOAT4), implicated in the activation of GHRL, is overexpressed in BC tissue samples^9^. Although cholecystokinin (CCK) has not been associated with the risk of BC, it has been reported that it affects the proliferation of pancreatic cancer cells^10^. Recently, in the Breast Cancer Health Disparities Study, we found associations between genetic variants of some of these genes and the risk of BC^11^.

Another key factor associated with BC development is insulin-like growth factor-1 (IGF-1) signaling. IGF-1 regulates fuel metabolism and is an important regulator of cell growth, proliferation, survival, differentiation and cellular transformation in many types of cancer, including BC^12–14^. Insulin-like growth factor binding protein-3 (IGFBP-3) is the main protein that binds to IGF-1. IGFBP-3, by binding to IGF-1, increases the half-life of circulating IGF-1, modulating its availability and its mitogenic and anti-apoptotic effects^13^. In an independent manner, IGFBP-3 is able to regulate the survival and proliferative activity of healthy and cancer cells^15^. Several studies have shown that IGF-1 serum concentration is positively associated with BC risk^16–19^. The association between the serum concentration of IGFBP-3 and the risk of BC has been inconsistent. Some studies have reported positive associations^19–21^, while others have shown inverse relationships^21, 22^. At a genetic level, variants at 2q35 identified by genome wide association studies (GWAS) in European ancestry women may mediate their effect via IGFBP-5^23^.

Based on this evidence, we hypothesized that some energy homeostasis genes may modify the association of IGF-1 and IGFBP-3 with the risk of BC. Therefore, the aim of this study was to assess the modifying effect of genetic variants in 10 energy homeostasis genes (*ADIPOQ, CART Prepropeptide* (*CARTPT), CCK, GHRL*, *LEP*, *LEP receptor* (*LEPR), MBOAT*, *melanocortin-4-receptor gene* (*MC4R*), *NPY* and *proopiomelanocortin* (*POMC*)) on the association of serum concentrations of IGF-1 and IGFBP-3 with the risk of BC.

## Results

The mean age of premenopausal cases in women from the 4-CBCS and MBCS was 44.3 years and 42.9 years, respectively; the mean age for controls was 42.5 and 42.2, respectively. BMI was higher in controls than in cases and was higher in women from the MBCS than in those from the 4-CBCS. Energy intake was higher in cases than in controls and was higher in women from the 4-CBCS compared with those from the MBCS. Serum concentrations of IGF-1 and IGFBP-3 were higher in cases than in controls in both studies. IGF-1 was higher in both cases and controls in the MBCS; in contrast, IGFBP-3 was higher in both cases and controls in women from the 4-CBCS (Table 1).

**Table 1:** Subject characteristics by study (premenopausal women), the Breast Cancer Health Disparities Study.

Characteristics	4-Corner’s Breast Cancer Study (4-CBCS)		Mexico Breast Cancer Study (MBCS)	
	Control	Case	Control	Case
	n = 155	n = 61	n = 282	n = 204
Age (years)^a^	42.5 (6.5)	44.3 (5.0)	42.2 (4.5)	42.9 (5.1)
Age at menarche (years)^a^	12.8 (1.6)	13.1 (1.7)	12.7 (1.7)	12.7 (1.5)
Genetic ancestry (% Indigenous)^a^	19.7 (22.6)	16.3 (22.9)	71.1 (17.4)	69.1 (18.0)
BMI (Kg/m2)^a^	28.4 (6.7)	26.4 (6.4)	30.0 (5.4)	28.3 (4.9)
Height (cm)^a^	161.9 (7.3)	161.7 (7.5)	153.4 (5.5)	154.5 (5.5)
Energy intake (Kcal/day)^a^	2322.5 (994.7)	2500.4 (1092.9)	2023.4 (729.6)	2232.7 (755.0)
IGF-1 serum concentration (ng/mL)^a^	145.9 (50.0)	152.3 (42.9)	171.2 (98.7)	224.7 (101.5)
IGFBP-3 serum concentration (ng/mL)^a^	4241.4 (761.6)	4455.0 (611.2)	3158.3 (999.5)	4160.5 (1153.4)
Ever use Oral Contraceptives (yes)^b^	69.7	78.0	53.9	57.4
Ever alcohol consumption (yes)^b^	41.8	47.5	1.8	3.9
Ever cigarette smoking (yes)^b^	28.4	31.2	22.7	27.9
First-degree family history of breast cancer (yes)^b^	16.1	18.3	4.3	5.4
**Parity**^**b**^				
Nulliparous	18.1	16.4	7.1	5.9
1 or 2	43.9	47.5	40.1	45.1
3 or more	38.1	36.1	52.8	49.0
**Education**^**b**^				
Low	7.1	4.9	85.5	76.5
Middle	17.4	18.0	8.2	13.2
High	75.5	77.1	6.4	10.3
**IGF-1 serum concentration (ng/mL)**^**b**^^**c**^				
Tertile 1	33.6	27.9	33.3	17.2
Tertile 2	33.6	31.2	33.7	25.0
Tertile 3	32.9	41.0	33.0	57.8
**IGFBP-3 serum concentration (ng/mL)**^**b**^^**d**^				
Tertile 1	33.6	21.3	33.3	8.8
Tertile 2	33.6	34.4	33.3	29.4
Tertile 3	32.9	44.3	33.3	70.6

^a^ Mean and standard deviation (SD).

^b^ Percentage.

^c^ Cut points of tertiles of serum IGF-1 in 4-Corner’s Breast Cancer Study were (122.0, 161.0); in Mexico Breast Cancer Study (123.6, 200.7).

^d^ Cut points of tertiles of serum IGFBP-3 1 in 4-Corner’s Breast Cancer Study were (3916, 4490); in Mexico Breast Cancer Study (2744, 3535).

For this study, 78 SNPs from 10 energy homeostasis genes were included (Supplementary Table S1); all of them had a minor allele frequency higher than 1%. After data from the 4-CBCS and MBCS populations were harmonized, HWE was tested in controls from both studies. Three SNPs (*LEPR* rs6673324, and rs1137101; *POMC* rs6713532) in women from the 4-CBCS (Supplementary Table S2) and four SNPs (*CARTPT* rsrs2239670; *LEPR* rs9436739, and rs1938484; *NPY* rs2023890) from the MBCS were not in HWE (Supplementary Table S3). These SNPs were not included in the analysis. The comparison of genotype frequencies between cases and controls in the 4-CBCS showed statistically significant differences (*P* < 0.05) in two genes: *ADIPOQ* (rs3821799 and rs1063537) and *LEP (*rs3828942) (Supplementary Table S2). In the MBCS, differences were observed in *LEPR* (rs12145690) (Supplementary Table S3). The genotype frequency comparison between studies showed significant differences for SNP rs3774261 in the *ADIPOQ* gene (Supplementary Table S3).

## Energy homeostasis genes modified the association of serum concentrations of IGF-1 and IGFBP-3 with BC risk

### IGF-1

The unadjusted analysis of the association between IGF-1 serum concentration and the risk of BC showed that compared to women in the lowest tertile, those in the highest tertile had a statistically significant increased risk of BC (OR = 2.79; 95% CI 1.88, 4.13) (Table 2).

**Table 2:** Association between serum concentration of IGF-1 and breast cancer by SNPs of genes regulating energy homeostasis, the Breast Cancer Health Disparities Study.

**IGF-1 association with premenopausal breast cancer**												
Variables	OR	95% CI	*P *value									
IGF-1 T1	1.00											
IGF-1 T2	1.34	0.87, 2.04	0.18									
IGF-1 T3	2.79	1.88, 4.13	< 0.0001									

**Interaction Co-dominant models**												
											IGF-1*SNP *P*_interaction_	
SNP	Variables	OR	95% CI	*P* value	OR	95% CI	*P* value	OR	95% CI	*P* value	Raw	Adjusted^a^
***ADIPOQ***	0 = GG; Cases = 223; Controls = 338				1 = GA; Cases = 26; Controls = 49			2 = AA; Cases = 0; Controls = 2				
rs17366568	IGF-1 T1	1.00			1.00							
0 = GG/1 = GA 2 = AA	IGF-1 T2	0.96	0.57, 1.63	0.89	0.14	0.01, 1.54	0.11					
n = 561/n = 75/n = 2	IGF-1 T3	1.62	0.93, 2.82	0.09	0.09	0.01, 0.91	**0.04**				0.098	0.944
***GHRL***	0 = TT; Cases = 155; Controls = 234				1 = TC; Cases = 83; Controls = 126			2 = CC; Cases = 11; Controls = 29				
rs27647	IGF-1 T1	1.00			1.00							
0 = TT /1 = TC 2 = CC	IGF-1 T2	0.96	0.50, 1.84	0.89	0.70	0.29, 1.71	0.44					
n = 389/n = 209/n = 40	IGF-1 T3	1. 75	0.90, 3.42	0.10	1.13	0.45, 2.86	0.79				0.039	0.312
***LEPR***	0 = TT; Cases = 90; Controls = 140				1 = TG; Cases = 119; Controls = 193			2 = GG; Cases = 40; Controls = 56				
rs970468	IGF-1 T1	1.00			1.00			1.00				
0 = TT /1 = TG/2 = GG	IGF-1 T2	0.71	0.30, 1.69	0.44	0.95	0.45, 1.97	0.88	1.24	0.29, 5.29	0.77		
n = 230/n = 312/n = 96	IGF-1 T3	1.52	0.61, 3.79	0.37	1.42	0.67, 3.02	0.36	0.59	0.13, 2.77	0.50	0.089	0.587
	0 = GG; Cases = 201; Controls = 303				1 = GA; Cases = 42; Controls = 83			2 = AA; Cases = 6; Controls = 3				
rs12059300	IGF-1 T1	1.00			1.00							
0 = GG/1 = GA 2 = AA	IGF-1 T2	0.83	0.46, 1.49	0.54	0.96	0.31, 3.04	0.95					
n = 504/n = 125/n = 9	IGF-1 T3	1.48	0.82, 2.68	0.19	0.61	0.15, 2.44	0.49				0.003	0.069
***MBOAT4***	0 = AA; Cases = 53; Controls = 99				1 = AG; Cases = 120; Controls = 176			2 = GG; Cases = 76; Controls = 114				
rs13272159	IGF-1 T1	1.00			1.00		1.00	1.00				
0 = AA/1 = AG/2 = GG	IGF-1 T2	3.92	0.91, 16.97	0.07	0.60	0.27, 1.32	0.60	0.89	0.37, 2.18	0.80		
n = 152/n = 296/n = 190	IGF-1 T3	7.16	1.64, 31.25	**0.009**	1.29	0.57, 2.94	1.29	0.60	0.23, 1.59	0.31	**0.027**	**0.027**
***NPY***	0 = AA; Cases = 214; Controls = 332				1 = AG; Cases = 32; Controls = 53			2 = GG; Cases = 3; Controls = 4				
rs16131	IGF-1 T1	1.00			1.00							
0 = AA/1 = AG 2 = GG	IGF-1 T2	1.14	0.66, 2.00	0.64	0.13	0.03, 0.71	**0.02**					
n = 546 /n = 85/n = 7	IGF-1 T3	1.77	1.00, 3.14	0.05	0.28	0.05, 1.64	0.16				**0.004**	**0.012**
***POMC***	0 = GG; Cases = 209; Controls = 340				1 = GA; Cases = 39; Controls = 48			2 = AA; Cases = 1; Controls = 1				
rs7565427	IGF-1T1	1.00			1.00							
0 = GG/1 = GA 2 = AA	IGF-1T2	1.20	0.70, 2.06	0.52	0.05	0.01, 0.41	**0.006**					
n = 549/n = 87/n = 2	IGF-1T3	1.89	1.07, 3.35	**0.03**	0.05	0.01, 0.51	**0.01**				0.079	0.316

The model at the top of the table was not adjusted for potential confounders. The rest of the models were adjusted for IGFBP-3 serum concentration (ng/mL), age (years), genetic ancestry (% Indigenous), BMI (Kg/m2), energy intake (Kcal/day), height (cm), age at menarche (years), ever use oral contraceptives (no,yes), parity (nulliparous, 1 or 2, 3 or more), study (4-CBCS, MBCS), first-degree family history of breast cancer (no, yes), ever alcohol consumption (no, yes) and ever cigarette smoking (no, yes). ^a^Benjamini-Hochberg adjusted *P* values (correction for the number of SNPs tested per gene); a threshold of 0.2 was considered significant.

We assessed the interaction between IGF-1 and the 71 SNPs and found an effect modification with a *P*_interaction_ < 0.05 for four SNPs in four genes (*GHRL*, *LEPR*, *MBOAT*, and *NPY*) and with a *P*_interaction_ from 0.05 to < 0.10 for three SNPs in three genes (*ADIPOQ*, *LEPR*, and *POMC*) in co-dominant models (Table 2). When comparing the effect of the highest IGF-1 tertile vs. the lowest, for most SNPs, there was a protective effect in heterozygous women; these effects were statistically significant for *ADIPOQ* rs17366568 (OR = 0.09; 95% CI 0.01, 0.91; *P* = 0.04), and *POMC* rs7565427 (OR = 0.05; 95% CI 0.01, 0.51; *P* = 0.01) (Table 2). Of particular interest, the SNP rs13272159 of *MBOAT4* showed an increased risk when comparing the effect of the highest IGF-1 tertile vs. the lowest (OR = 7.16; 95% CI 1.64, 31.25; *P* = 0.009) in women with AA; the SNP rs7565427 of *POMC* also showed an increased risk (OR = 1.89; 95% CI 1.07, 3.35; *P* = 0.03) in women with GG. After adjusting for multiple comparisons (FDR correction), only interactions for three SNPs in three genes (*LEPR* rs12059300, *MBOAT* rs13272159 and *NPY* rs16131) were statistically significant with an adjusted *P*_interaction_ < 0.20.

After assesing the association between the Table 2 SNPs and IGF-1 serum concentration, we found that for the LEPR rs970468 SNP, control women who were carriers of the GG variant had higher IGF-1 serum concentration (1.23 ng/mL, *P* = 0.010) than TT homozygotes. However, after adjusting for multiple comparisons, this association was not statistically significant (adjusted *P* > 0.05) (data not shown).

### IGFBP-3

The unadjusted analysis of the association between IGFBP-3 serum concentration and the risk of BC showed that compared to women in the lowest tertile, those in the highest tertile had a statistically significant increased risk of BC (OR = 5.55, 95% CI 3.55, 8.68) (Table 3).

**Table 3:** Association between serum concentration of IGFBP-3 and breast cancer by SNPs of genes regulating energy homeostasis, the Breast Cancer Health Disparities Study.

**IGFBP-3 association with premenopausal breast cancer**												
Variables	OR	95% CI	*P* value									
IGFBP-3 T1	1.00											
IGFBP-3 T2	2.03	1.25, 3.31	0.004									
IGFBP-3 T3	5.55	3.55, 8.68	< 0.001									

**Interaction Co-dominant models**												
											IGF-1*SNP *P*_interaction_	
SNP	Variables	OR	95% CI	*P* value	OR	95% CI	*P* value	OR	95% CI	*P* value	Raw	Adjusted^a^
***ADIPOQ***	0 = GG; Cases = 59; Controls = 109				1 = GA; Cases = 128; Controls = 194			2 = AA; Cases = 55; Controls = 83				
rs182052	IGFBP-3 T1	1.00			1.00			1.00				
0 = GG /1 = GA/2 = AA	IGFBP-3 T2	0.69	0.23, 2.07	0.51	3.11	1.39, 6.97	**0.006**	1.91	0.46, 7.93	0.37		
n = 168/n = 322/n = 138	IGFBP-3 T3	1.45	0.52, 4.08	0.48	4.92	2.15, 11.23	** < 0.001**	16.52	3.94, 69.26	** < 0.001**	**0.027**	**0.164**
	0 = TT; Cases = 173; Controls = 262				1 = TC; Cases = 70; Controls = 113			2 = CC; Cases = 6 ; Controls = 14				
rs822391	IGFBP-3 T1	1.00			1.00							
0 = TT/1 = TC 2 = CC	IGFBP-3 T2	2.73	1.37, 5.44	**0.004**	0.9	0.32, 2.49	0.83					
n = 435/n = 183/n = 20	IGFBP-3 T3	6.64	3.36, 13.14	** < 0.001**	2.61	0.91, 7.48	0.08				**0.041**	**0.164**
	0 = AA; Cases = 134; Controls = 211				1 = AT; Cases = 99 ;Controls = 145			2 = TT; Cases = 16; Controls = 33				
rs7649121	IGFBP-3 T1	1.00			1.00							
0 = AA/1 = AT 2 = TT	IGFBP-3 T2	1.51	0.73, 3.13	0.27	1.65	0.66, 4.17	0.29					
n = 345/n = 244/n = 49	IGFBP-3 T3	3.23	1.54, 6.77	**0.002**	5.63	2.28, 13.88	** < 0.001**				**0.036**	**0.164**
	0 = CC; Cases = 78; Controls = 116				1 = CT; Cases = 120; Controls = 191			2 = TT; Cases = 51; Controls = 82				
rs3821799	IGFBP-3 T1	1.00			1.00			1.00				
0 = CC/1 = CT /2 = TT	IGFB-P3 T2	2.12	0.71, 6.30	0.18	1.15	0.52, 2.55	0.73	2.92	0.86, 9.89	0.09		
n = 194/n = 311/n = 133	IGFBP-3 T3	11.73	3.72, 36.9	** < 0.001**	3.23	1.50, 6.93	**0.003**	3.22	0.88, 11.74	0.08	0.078	0.187
	0 = GG; Cases = 82; Controls = 131				1 = GA; Cases = 119; Controls = 184			2 = AA; Cases = 48; Controls = 73				
rs3774261	IGFBP-3 T1	1.00			1.00			1.00				
0 = GG/1 = GA /2 = AA	IGFBP-3 T2	2.32	0.82, 6.59	0.11	1.38	0.61, 3.09	0.44	2.30	0.64, 8.18	0.20		
n = 213/n = 303/n = 121	IGFBP-3 T3	10.80	3.74, 31.19	** < 0.001**	3.81	1.75, 8.27	**0.001**	2.24	0.57, 8.88	0.25	0.057	0.171
***CARTPT***	0 = GG; Cases = 198; Controls = 304				1 = GC; Cases = 45 ; Controls = 78			2 = CC; Cases = 6 ; Controls = 7				
rs3846659	IGFBP-3 T1	1.00			1.00							
0 = GG/1 = GC 2 = CC	IGFBP-3 T2	1.60	0.87, 2.96	0.13	2.97	0.72, 12.18	12.18	0.13				
n = 502/n = 123/n = 13	IGFBP-3 T3	5.89	3.22, 10.79	** < 0.001**	1.95	0.41, 9.26	9.26	0.40			**0.031**	**0.124**
***LEPR***	0 = GG; Cases = 201; Controls = 303				1 = GA; Cases = 42 ; Controls = 83			2 = AA; Cases = 6 ; Controls = 3				
rs12059300	IGFBP-3 T1	1.00			1.00							
0 = GG/1 = GA 2 = AA	IGFBP-3 T2	2.41	1.26, 4.6	**0.008**	1.01	0.26, 3.91	0.99					
n = 504/n = 125/n = 9	IGFBP-3 T3	6.25	3.22, 12.15	** < 0.001**		1.04, 12.02	**0.043**				**0.008**	**0.184**
	0 = GG; Cases = 206; Controls = 299				1 = GT; Cases = 39 ; Controls = 81			2 = TT; Cases = 4 ; Controls = 9				
rs11585329	IGFBP-3 T1	1.00			1.00							
0 = GG /1 = GT 2 = TT	IGFBP-3 T2	1.94	1.07, 3.51	**0.03**	0.77	0.14, 4.19	0.76					
n = 505/n = 120/n = 13	IGFBP-3 T3	4.12	2.28, 7.45	** < 0.001**	8.70	1.74, 43.56	**0.008**				0.075	0.849
***NPY***	0 = CC; Cases = 102; Controls = 165				1 = CA; Cases = 110; Controls = 169			2 = TT; Cases = 31; Controls = 48				
rs16141	IGFBP-3 T1	1.00			1.00			1.00				
0 = CC/1 = CA/2 = AA	IGFBP-3 T2	2.05	0.79, 5.36	0.14	1.72	0.75, 3.95	0.20	0.86	0.20, 3.81	0.85		
n = 267/n = 279/n = 92	IGFBP-3 T3	6.8	2.51, 18.4	** < 0.001**	4.48	1.96, 10.24	** < 0.001**	1.61	0.41, 6.30	0.50	0.056	0.116
	0 = GG; Cases = 110; Controls = 185				1 = GT; Cases = 108; Controls = 156			2 = TT; Cases = 31; Controls = 48				
rs16129	IGFBP-3 T1	1.00			1.00			1.00				
0 = GG/1 = GT/2 = TT	IGFBP-3 T2	2.23	0.89, 5.58	0.09	1.43	0.62, 3.27	0.40	1.09	0.21, 5.68	0.92		
n = 295/n = 264/n = 79	IGFBP-3 T3	6.40	2.52, 16.25	** < 0.001**	3.75	1.64, 8.56	**0.002**	2.41	0.58, 10.4	0.23	0.077	0.116

The model at the top of the table was not adjusted for potential confounders. The rest of the models were adjusted for IGF-1 serum concentration (ng/mL), age (years), genetic ancestry (% Indigenous), BMI (Kg/m2), energy intake (Kcal/day), height (cm), age at menarche (years), ever use oral contraceptives (no,yes), parity (nulliparous, 1 or 2, 3 or more), study (4-CBCS, MBCS), first-degree family history of breast cancer (no, yes), ever alcohol consumption (no, yes) and ever cigarette smoking (no, yes). ^a^Benjamini-Hochberg adjusted P value (correction for the number of SNPs tested per gene); a threshold of 0.2 was considered significant.

For IGFBP-3, we also assessed the interaction with the 71 SNPs and found an effect modification with a *P*_interaction_ < 0.05 for five SNPs in three genes (*ADIPOQ*, *CARTPT* and *LEPR*) and with a *P*_interaction_ from 0.05 to < 0.10 for five SNPs in three (*ADIPOQ, LEPR,* and *NPY*) out of the 10 genes included in the analysis (Table 3). In the codominant models, all SNPs except three (*ADIPOQ* rs182052, and rs7649121; and *LEPR* rs11585329) showed the same pattern. For seven SNPs (*ADIPOQ* rs822391, rs3821799, and rs3774261; *CARTPT* rs3846659; *LEPR* rs12059300; *NPY* rs16141 and rs16129), when comparing the effect of the highest IGFBP-3 serum concentration tertile vs. the lowest, there was a statistically significant increased BC risk in those who were homozygous for the major allele. This effect was lower in heterozygous, and the effect was not observed in women who were homozygous for the minor allele. For example, for *ADIPOQ* rs3774261 GG genotype, we observed an OR = 10.80 (95% CI 3.74, 31.19; *P* < 0.001); for the GA genotype, an OR = 3.81 (95% CI 1.75, 8.27; *P* = 0.001); and for the AA genotype, an OR = 2.24 (95% CI 0.57, 8.88; *P* = 0.25) (Table 3). All interactions except (LEPR rs11585329) remained statistically significant with an adjusted *P*_interaction_ < 0.20 after adjusting for multiple comparisons (FDR correction).

After assessing the association between the Table 3 SNPs and IGFBP-3 serum concentration, we found that in control women, there was an association between 4 *ADIPOQ* SNPs (rs7649121, rs182052, rs3774261, rs3821799) and the IGFBP-3 serum concentration (*P* < 0.05). However, after adjusting for multiple comparisons, only the association for rs7649121 remained statistically significant. Compared to AA homozygotes TT carriers had a lower IGFBP-3 serum concentration (-710.1 ng/mL) (*P* =  < 0.001) (data not shown).

## Discussion

In this study, we showed that variants of some energy homeostasis genes modified the association between IGF-1 or IGFBP-3 serum concentration and the risk of BC in premenopausal women. Several epidemiological studies have highlighted the existing association between IGF-1 and IGFBP-3 serum concentration and the risk of BC^12, 16, 19, 21, 22^. IGF-1 and IGFBP-3 regulate multiple biological mechanisms, including fuel metabolism and cell growth, proliferation, migration, metastasis and angiogenesis^15, 24^. IGF-1 activates downstream pathways involved in carcinogenic processes such as the PI3K/AKT, RAS/RAF/MAPK, and STAT cascades^24^. IGFBP-3, in addition to modulating IGF-1 activity, may also trigger IGF-independent activation of various signaling pathways, including MAPK, AKT, Smad and STAT^15, 25^. Recently, we reported an association between some SNPs of energy homeostasis genes and the risk^11^ or mortality^26^ of BC. Other authors have also reported associations between variants of these genes and BC risk^27–29^. As we described below, evidence shows that some energy homeostasis genes may also regulate signaling pathways involved in cancer development, supporting their potential to modify the association between IGF-1 and IGFBP-3 and the risk of BC.

In our study, seven SNPs in five genes (*MBOAT4* rs13272159, *NPY* rs16131, *ADIPOQ* rs182052, rs822391 and rs7649121, *CARTPT* rs3846659, and *LEPR* rs12059300) showed a significant modifying effect (*P*_interaction_ < 0.05; adjusted *P*_interaction_ < 0.20), with at least one tertile of IGF-1 or IGFBP-3 associated with the risk of BC (*P* value < 0.05).

We found that one SNP of *MBOAT4* significantly modified the association of IGF-1 serum concentration and the risk of BC in premenopausal women. For the intronic (3'UTR) SNP of *MBOAT4*, rs13272159, the positive effect of the highest tertile of IGF-1 on the risk of BC was observed in women with the AA genotype but not in the presence of the G allele. There is evidence that supports the possible role of *MBOAT4* in BC. The *MBOAT4* gene encodes GHRL O-acyltransferase (GOAT), an enzyme that catalyzes GHRL octanoylation regulating GHRL activity^30^. The regulation of growth hormone (GH) and IGF-1 release is under the influence of GHRL^27, 30^. In our study, we did not find an association between the *MBOAT4* rs13272159 SNP and the serum concentration of IGF-1 (data not shown). The peptide hormone GHRL, in addition to having a role in the regulation of feeding and energy balance, also regulates processes associated with cancer, such as cell proliferation, apoptosis, cell migration, cell invasion, angiogenesis and inflammation. The GHRL proliferation effect could be mediated through the activation of key signaling pathways, such as MAPK/ERK and/or PI3K/AKT/mTOR^8, 9, 30^. Although a precise role for GHRL in BC has not yet been established, it has been suggested that an imbalance in the expression of the GHRL system in mammary tissue could be implicated in breast tumor pathogenesis^9^. *MBOAT4* mRNA expression seems to be upregulated in BC tumors^9^. Our group and other authors have reported an association between *GHRL* SNPs and BC risk^11, 28, 31^ or BC-specific mortality^26^. In addition, in a recent cohort study, GHRL expression was associated with increased survival in node-negative patients^32^.

For the intronic SNP of *NPY*, rs16131, the highest tertile of IGF-1 concentration was borderline associated with an increased risk of BC in the presence of AA but not in G carriers. The central orexigenic regulator NPY and its receptors have been implicated as growth-promoting factors in various cancer types. It has been shown that NPY regulates proliferation, migration, and vascular endothelial growth factor (VEGF) release to promote angiogenesis in BC cells. The mitogenic effects of NPY seem to be mediated by the p44/42 MAPK pathway in some malignancies^3^. It has been suggested that NPY may influence GH release (by modulating growth hormone-releasing hormone (GHRH) secretion)^33^, thus modulating IGF-1 release. There was no association between the *NPY* rs16131 SNP and the serum concentration of IGF-1 (data not shown). NPY receptor type 1 (NPY1R) has been proposed as a novel peripheral blood marker predictive of metastasis and prognosis in patients with BC^34^. In a previous work, we found that another *NPY* SNP, rs16129, was significantly associated with BC-specific mortality; however, no effect was observed for the rs16131 SNP^26^.

Our results showed that the association between IGFBP-3 serum concentration and BC risk differed by *ADIPOQ* SNPs (rs822391, rs7649121 and rs182052). In the case of *ADIPOQ* SNP rs822391, an increase in BC risk was observed for the highest tertile of serum concentration of IGFBP-3 in TT carriers and was lost in the presence of the minor allele C. Interestingly, other authors have reported that rs822391 (minor allele C) decreases the risk of overall prostate cancer^35^. Regarding the *ADIPOQ* rs7649121 SNP, compared to women in the lowest IGFBP-3 serum concentration tertile, women in the highest tertile showed higher BC risk. This association was higher in carriers of the T allele, than in AA homozygotes. To our knowledge, no association between this SNP and the risk of any cancer has been previously reported. For *ADIPOQ* SNP rs182052, our data showed that the positive effect of the highest tertile of IGFBP-3 concentration on the risk of BC was higher in the presence of the minor allele A. These results are consistent with other studies that have found that rs182052 (minor allele A) increased the risk of other types of cancer^35, 36^. Furthermore, these studies observed an association between rs182052 (minor allele A) and low serum and plasma ADIPOQ concentrations^35, 36^, suggesting a biological causal link. Epidemiological studies have found that ADIPOQ plasma concentrations are inversely associated with the risk of several malignancies, including BC^7^. It has been suggested that ADIPOQ is a BC malignant progression and prognosis biomarker^6^. Research on ADIPOQ has provided evidence for the pleiotropic role of this hormone; it regulates insulin-sensitizing, inflammation, and tumoral actions^37^. Some studies have shown that ADIPOQ regulates GH secretion from rat pituitary cells in vitro, both positively^38^ and negatively^39^. In addition, ADIPOQ receptors are expressed in human pituitary GH-producing cells^40^, suggesting that alterations in ADIPOQ expression could affect GH serum concentration and consequently IGF-1 and IGFBP-3 release. In our study, there was a statistically significant association between the *ADIPOQ* rs7649121 SNP and a lower serum concentration of IGFBP-3. The ADIPOQ induces cell growth arrest and apoptosis and suppresses cell proliferation, invasion and migration in ER-negative BC cells, whereas controversial observations have been reported for ER-positive BC cells^5^. ADIPOQ is known to block proliferative signals of IGF-1 and LEP^6^. ADIPOQ signaling induces AMPK activation and consequent inhibition of the MAPK, PI3K/Akt, WNT-β-catenin, NFκB, and JAK2/STAT3 pathways^5, 7^. Given that the ADIPOQ rs7649121 SNP was associated with the IGFBP-3 serum concentration it is not possible to disentangle if the modifying effect was due to a breast cancer signaling pathway or to the effect of the SNP on the IGFBP-3 serum concentration.

We also found an effect modification on the association between the IGFBP-3 serum concentration and the risk of BC by *LEPR* SNP (rs12059300). Our results show that the positive effect of the IGFBP-3 highest serum concentration tertile on the BC risk observed in women with the GG decreased in the presence of the minor allele A. Interestingly, it has been reported that rs12059300 interacts with methylation sites in the 5´UTR of *LEPR,* influencing LEP serum levels (LEP levels decrease for AA compared to the GG genotype)^41^. Some studies have observed an association between high LEP plasma concentrations and BC risk^5, 7^. High LEP serum concentrations and LEPR overexpression have been positively correlated with the reduction in overall survival rates in patients with BC^6^. In a previous work, using the samples of the present study plus samples from other centers, we found that some *LEPR* SNPs were associated with BC risk^11^ and with BC mortality^26^ within specific ancestry strata; however, there was no association between rs12059300 and BC risk or mortality^11, 26^. Experimental data obtained with LEPR knockout mice suggested that LEP signaling could induce GH release^42^, thus regulating not only IGF-1 but also IGFBP-3 release. We did not find an association between the *LEPR* rs12059300 SNP and the IGFBP-3 serum concentration (data not shown). In addition to its neuroendocrine role, LEP and LEPR regulate a wide range of biological processes, including mammary tumorigenesis^5, 7^. LEP signaling promotes cell proliferation, differentiation, survival, migration, and invasion via the activation of downstream pathways, including the JAK2/STAT3, MAPK and PI3K/Akt pathways^5, 7^. LEP crosstalk has also been reported with estrogen receptor alpha (ERα) via ERα transactivation^5^ and the IGF-1 pathway through mTORC1 and STAT5^43^. Based on these studies, our results suggest that the *LEPR* rs12059300 minor allele A could contribute to the reduction in BC risk. Notably, in addition to its modifying effect on the association between IGFBP-3 and BC risk, rs12059300 also showed an effect modification on the association between IGF-1 and the risk of BC (*P*_interaction_ < 0.05; adjusted *P*_interaction_ < 0.20); however, this was not discussed in the IGF-1 section because associations inside strata were not statistically significant.

Finally, regarding the *CARTPT* SNP rs3846659, in women with the GG genotype, compared to the lowest IGFBP-3 serum concentration tertile, women in the highest tertile showed an increased BC risk, but this effect was lost in the presence of the minor allele C. In a previous work, our group found that rs3846659 was associated with the risk of premenopausal BC^11^. *CARTPT* encodes CART prepropeptide, which ends up generating multiple biologically active peptides that have a role in the regulation of appetite, energy balance, maintenance of body weight, reward and addiction, and the stress response^44, 45^. CART is involved in the pituitary hormone secretion. Intracerebroventricular or peripheral administration of CART increases the GH concentration in rats^45^, suggesting that *CARTPT* variants may modify the synthesis of IGF-1 and IGFBP-3. In our study, we did not find an association between the *CARTPT* rs3846659 SNP and the serum concentration of IGFBP-3 (data not shown). CART is expressed in primary and metastatic breast cancer; mediates ligand-independent activation of ERα through the MAPK pathway; and is an independent poor prognostic factor in ER-positive, lymph node-negative BC tumors^4^.

We were able to combine data from two population-based case–control studies conducted in the United States (4-Corner’s Breast Cancer Study) and Mexico (Mexico Breast Cancer Study). Given that we used similar questionnaires for both studies, we were able to harmonize the lifestyle data. A tag SNP approach was used to characterize variation across candidate genes. This approach was implemented on a customized Illumina platform and included SNPs that were validated and considered to have a high probability of yielding results. We adjusted for multiple comparisons to identify SNPs with a relevant modifying effect, however spurious interactions cannot be excluded.

Regarding the limitations, only premenopausal women were included in this study. Previous large epidemiological studies have found statistically significant associations between IGF-1 and breast cancer only in premenopausal women; in postmenopausal women the associations were weaker and not statistically significant^46–48^. The percentage of indigenous ancestry was higher for the MBCS women than for those included in the 4-CBCS study. Therefore, we adjusted for the effect of both the study and indigenous ancestry in all multiple models. IGF-1 and IGFBP-3 serum concentrations were measured in two different labs using two different assays. Some of the IGF-I and IGFBP-3 serum concentration discrepancies (less IGF-1 and more IGFBP-3 in the 4-CBCS than in the MBCS) between MBCS and 4-CBCS studies could be related to assay methodology or to differences in characteristics between women from both studies. More women in the 4-CBCS reported to consume alcohol than in the MBCS (controls = 41.8%, cases 47.5%; controls = 1.8%, cases = 3.9%, respectively). Alcohol consumption has shown to reduce IGF-I serum concentration and to increase IGFBP-3 serum concentration^49^. Although the IGF-1/IGBP-3 ratio may vary between studies (0.03 to 0.10)^50–52^; the ratio remained constant in both cases and controls. In our study, the ratio in the 4-CBCS sample was 0.03 for both cases and controls, while in the MBCS sample it was 0.05 in both cases and controls. Therefore, we consider that it is unlikely that these discrepancies influenced our results. Although evidence suggest that carrying a BRCA mutation could be a potential confounder of the association between IGF-1 serum concentration and BC risk^53, 54^, we did not exclude women with these mutations, because we have this information only for the MBCS women with breast cancer and not for controls. The prevalence of this mutation in the MBCS cases was 4.2%, therefore adjusting for women with this mutation, could have not significantly altered our results. Finally, there is no information about the functional effect on protein expression caused by several of the SNPs analyzed in this study, thus limiting the biological comprehension of the observed interactions.

In summary, our data show for the first time that variants of energy homeostasis genes modify the effect of IGF-1 or IGFBP-3 serum concentration on BC risk in premenopausal women. We found that *MBOAT4* and *NPY* SNPs modified the association between IGF-1 serum concentration and the risk of BC, and *ADIPOQ*, *LEPR* and *CARTPT* SNPs modified the association between IGFBP-3 and the risk of BC. Our data suggests that these genes could act as modifiers through their influence on known signaling pathways involved in BC development. Our results should be replicated in longitudinal studies and the molecular mechanisms responsible for the effect modifications that we found need to be further investigated. This study provides data that supports and highlights the importance of analyzing the interplay between multiple risk factors to contribute to a better understanding of breast cancer etiology and, in the future, improving risk prediction.

## Methods

### Study population

As part of the Breast Cancer Health Disparities study^19^, the study population included premenopausal women from the 4-Corner’s Breast Cancer Study (4-CBCS)^55^ and the Mexico Breast Cancer Study (MBCS)^56^. The study population for the present study was restricted to premenopausal women because in longitudinal studies, the association between the serum concentration of IGF-1 and the risk of BC has been observed in pre- but not postmenopausal women^48, 57^. Briefly, the 4-CBCS included women living in Arizona, Colorado, New Mexico or Utah. Eligible cases were between 25 and 79 years old and were histologically confirmed between 1999 and 2004 (n = 2557), while controls (n = 2605) were selected from the target population and were frequency-matched to cases on the expected ethnicity and over a 5-year age range^19^. Interviews were performed by certified personnel^58^, blood was collected, and DNA was extracted for 79% of the participants; height, weight and waist and hip circumferences were obtained at interview^19^. For the present study, a subsample of 61 cases and 155 controls were included because they had fasting IGF-1 and IGFBP-3 measurements and were not receiving chemotherapy^52^. The MBCS included 1000 cases and 1074 controls between 35 and 69 years of age living in Monterrey, Veracruz and Mexico City for the past 5 years^56^. Eligible cases were women with BC histologically confirmed between January 2004 and December 2007. Controls were randomly selected from the catchment area of the 12 participating hospitals using a probabilistic multistage design. Controls were frequency-matched to cases, according to 5-year age groups, membership to a health care institution and place of residence. Interviews were performed by standardized personnel using a questionnaire based on the 4-CBCS. Blood was collected, and DNA was extracted from 91% of women. MBCS anthropometric measurements were obtained by standardized nurses at the hospitals. For this study, a random subsample of 204 cases and 282 controls who had serum IGF-1 and IGFBP-3 measurements was included.

### Serum biomarkers

For both studies, serum levels of IGF-1 and IGFBP-3 were measured in fasting blood samples^19, 52^. For the 4-CBCS, IGF-1 and IGFBP-3 serum concentrations were measured at the Maine Center for Osteoporosis Research as described elsewhere^59^. Briefly, serum IGF-1 serum concentrations were determined using the IGF-1 (IGFBP-3 blocked) radioimmunoassay (American Laboratory Products Company (ALPCO) Windham, NH), while IGFBP-3 serum concentrations were ascertained using the ‘‘Active’’ IGFBP-3 IRMA kit (Diagnostic Systems Laboratories, Inc., Webster, Texas). All samples were tested in replicate, and the coefficient of variation ranged from 0.06 to 6.5% for IGF-1 and 0.17 to 7.5% for IGFBP-3. For the MBCS, **serum** samples were frozen at temperatures between – 20 and -70 °C and processed no later than 4 weeks after the blood was collected. The IGF-1 and IGFBP-3 serum concentrations were determined using radioimmunoanalysis at Laboratorios Clínicos de Puebla, México (ISO 9001:2008).

### Data harmonization

Data were harmonized as described in Slattery (2012)^19^. Variables considered as potential confounders were age (years), genetic ancestry (% Indigenous), body mass index (BMI, kg/m^2^), energy intake (kcal/day), height (cm), age at menarche (years), ever used oral contraceptives (no, yes), parity (nulliparous, 1 or 2, 3 or more), education (low, middle, high), study (4-CBCS, MBCS), first-degree family history of breast cancer (no, yes), ever consumed alcohol (no, yes), ever smoked cigarettes (no, yes) and IGFBP-3 serum concentration (ng/mL) when IGF-1 was the independent variable of interest, or IGF-1 serum concentration (ng/mL) when IGFBP-3 was the independent variable of interest. Weight used to calculate the BMI was measured by a nurse at the time of diagnosis for the MBCS and was either self-reported weight during the referent year or weight reported five years prior to diagnosis if the referent year weight was not available for the 4-CBCS. Height was measured at the time of interview.

### Genetic data

DNA was derived from whole blood from both the 4-CBCS and the MBCS participants. Genotyping was conducted as part of the Breast Cancer Health Disparities Study^19^. European and Native American Ancestry were measured based on 104 ancestral informative markers. For this study, we evaluated 10 genes that regulate energy homeostasis: *ADIPOQ* (12 SNPs), *CARTP* (5 SNPs), *CCK* (4 SNPs), *GHRL* (8 SNPs), *LEP* (9 SNPs), *LEPR* (27 SNPs), *MBOAT4* (1 SNP), *MC4R* (3 SNPs), *NPY* (4 SNPs), and *POMC* (5 SNPs). Supplementary Table S1 describes the 78 SNPs in detail.

### Statistical analysis

A descriptive analysis of the study population was conducted. Means and standard deviations were used for continuous variables and percentages for categorical variables. Allele and genotype frequencies were obtained through direct counting. Hardy–Weinberg equilibrium (HWE) was tested by the exact test among control subjects. Comparison of genotype frequencies between cases and controls was adjusted for ancestry (% Indigenous) by means of logistic regressions; *P* values were obtained by using likelihood ratio tests. We used logistic regressions and likelihood ratio tests to compare ancestry adjusted genotype frequencies in control subjects from both populations. A *P* value < 0.05 was considered statistically significant. To evaluate the potential effect modification of the association between serum concentration of IGF-1 (tertiles) or IGFBP-3 (tertiles) and the risk of BC by energy homeostasis gene polymorphisms, multiple logistic regression models were performed. Associations with SNPs were assessed assuming a codominant model. Variables considered as potential confounders were described in the paragraph on data harmonization. Effect modification models are presented when *P* for the interaction was lower than 0.10. In addition, for multiple hypothesis testing correction Benjamini–Hochberg adjusted *P* values were calculated for the number of SNPs tested per gene^60^; a threshold of 0.20 was considered significant^61, 62^. The association between SNPs and IGF-1 and IGFBP-3 serum concentrations were analyzed. The Benjamini–Hochberg adjusted *P* values were calculated for the number of SNPs tested per gene; a *P* value < 0.05 was considered statistically significant. Logistic regression analyses were performed using Stata12 (Stata Corporation, College Station, TX) and false discovery rate adjustments were performed using R version 3.6.2.

All participants signed informed written consent prior to participation; the study was approved by the Institutional Review Board for protection of human subjects at each institution.

### Ethics approval

The study was approved by the Institutional Review Board for protection of human subjects at the National Institute of Public Health in Mexico and The University of Utah: “Comité de Ética en Investigación and Institutional Review Board, respectively” and was performed in accordance with the Declaration of Helsinki.

### Consent to participate

Signed informed consent of all women participating in the study was obtained.

### Consent for publication

All authors consent to the publication of this manuscript.

## Supplementary Information

Supplementary Information.

## Data Availability

The datasets generated during and/or analyzed during the current study are not publicly available because it is not a requirement at our institution but are available from the corresponding author on reasonable request.
